# Two Cases of *MYRF*-Related Differences of Sex Development: A Wide Phenotypic Spectrum

**DOI:** 10.1210/jcemcr/luaf277

**Published:** 2025-12-03

**Authors:** Allie Dayno, Prerana Chatty, Sara Muneer, Carolina Montano, Marissa Kilberg, Maria Vogiatzi

**Affiliations:** Children's Hospital of Philadelphia, 3501 Civic Center Blvd, Philadelphia, PA 19104, USA; Children's Hospital of Philadelphia, 3501 Civic Center Blvd, Philadelphia, PA 19104, USA; Children's Hospital of Philadelphia, 3501 Civic Center Blvd, Philadelphia, PA 19104, USA; Children's Hospital of Philadelphia, 3501 Civic Center Blvd, Philadelphia, PA 19104, USA; Children's Hospital of Philadelphia, 3501 Civic Center Blvd, Philadelphia, PA 19104, USA; Children's Hospital of Philadelphia, 3501 Civic Center Blvd, Philadelphia, PA 19104, USA

**Keywords:** *MYRF*, differences of sex development, gonadal failure

## Abstract

*MYRF*-related disorder is a recently described rare etiology of differences of sex development (DSD) caused by pathogenic variants in the *MYRF* gene on chromosome 11. *MYRF* encodes a membrane-bound transcription factor that is important in the early development of the eyes, lungs, diaphragm, heart, and genitourinary tract. With approximately 60 cases described, there is a need to better understand the associated phenotypes. We present 2 cases of 46, XY individuals found to have critical congenital cardiac disease prenatally, prompting genetic testing and, ultimately, variants in the *MYRF* gene to highlight the different phenotypes that can be seen in this disorder. Patient 1 had normal female genitalia, müllerian structures on imaging, and gonadal failure on biochemical testing. Conversely, patient 2 had perineal hypospadias, severe chordee, bifid scrotum, and bilateral cryptorchidism with normal testicular function in the mini-puberty period. Notably, both *MYRF* variants are novel and have not been previously reported in the medical literature, expanding the mutational and phenotypic spectrum of this condition.

## Introduction

Differences of sex development (DSD) are heterogeneous conditions of the reproductive system, characterized by discordance between chromosomal, gonadal, and anatomical sex [1]. DSD encompass a wide phenotypic spectrum and have various etiologies including sex chromosome abnormalities, variants in genes involved in gonadal or genital development, and disorders of steroidogenesis. While hormonal testing and karyotype can help narrow the differential, specific genetic testing is often necessary to elucidate the molecular diagnosis. Conventional testing includes chromosomal analysis, microarrays, and targeted sequencing panels. However, this approach is limited, with only 35% to 45% of individuals with 46, XY DSD receiving a molecular diagnosis [2-4]. Pathogenic variants in genes such as *NR5A1*, *SRDA52, AR*, and *DHH* have been identified as monogenic causes of DSD. Recently, broader next-generation sequencing (NGS) technologies, such as exome (ES) and genome sequencing (GS), offer faster, more comprehensive diagnostics for DSD, aiding the discovery of novel variants.

The *MYRF* gene (OMIM 608329) is on chromosome 11 and encodes a membrane-bound transcription factor called myelin regulatory factor (MYRF) that was originally identified as a transcription regulator of myelin in the central nervous system [5]. Since then, the MYRF protein has also been shown to influence early development of the diaphragm, heart, eyes, lungs, and genitourinary (GU) tract [6, 7]. Specifically, MYRF is expressed in coelomic epithelium of fetal gonads that forms the germinal epithelium. Variants in the *MYRF* gene have been identified as a cause of DSD by using NGS technology. Pathogenic variants in *MYRF* lead to *MYRF*-related ophthalmic cardiac urogenital syndrome (OCUGS), known as *MYRF*-CUGS or *MYRF*-OCUGS, which is a rare condition with autosomal dominant inheritance. The prevalence is unknown, with approximately 60 identified individuals so far [7].

As more genetic and clinical information emerges about *MYRF*-OCUGS, recognizing this entity as a potential cause of 46, XY DSD (and possibly 46, XX ovarian failure) and the associated phenotypes becomes increasingly important [6]. The various clinical phenotypes include urogenital differences (cryptorchidism, hypospadias, ambiguous genitalia, sex reversal), congenital heart disease, pulmonary hypoplasia, diaphragm anomalies, and nanophthalmos/high hyperopia [6, 7]. The presentation can vary greatly from isolated urogenital differences to severe cardiopulmonary disease. While the phenotype often depends on the location and functional consequences of the variant, no consistent genotype-phenotype correlation has been established besides the association between C-terminal pathogenic variants and eye findings [7]. Fig. 1 from the literature review by Zhang et al [6] illustrates the structure of the MYRF protein along with the distribution of reported pathogenic variants. Here, we discuss 2 cases of children with 46, XY DSD and novel *MYRF* variants who presented with different phenotypes, highlighting the wide spectrum of clinical manifestations associated with this condition in the newborn period and support the need for long-term monitoring of gonadal function.

## Case Presentation

### Case 1

Endocrinology was consulted for evaluation of female external genitalia in a 2-day-old ex 39-week newborn with prenatal testing showing a 46, XY chromosomal complement. The baby was also prenatally diagnosed with critical congenital cardiac disease (suspected scimitar syndrome). Evaluation after birth was significant for heterotaxy syndrome, dextrocardia, scimitar syndrome, severely hypoplastic right lung, interrupted inferior vena cava, transverse aortic arch hypoplasia, small apical muscular ventricular septal defect, atrial septal defect, and patent ductus arteriosus. Relevant to this report, the genital examination was notable for typical-appearing female external genitalia (Prader scale 0). Vaginal opening and urethra were visualized. There was no rugation of the labia majora and there were no palpable gonads on examination.

### Case 2

Endocrinology was consulted for evaluation of undervirilized genitalia in this 5-day-old ex 34-week infant with prenatal testing demonstrating 46, XY chromosome complement. Prenatally, the patient was found to have scimitar syndrome and severe hypospadias on ultrasound. Additional postnatal findings included moderate atrial septal defect, pulmonary hypoplasia, right congenital diaphragmatic hernia (CDH), and bilateral peripheral cataracts. The GU examination was significant for perineal hypospadias, severe chordee (>45° angle), bifid scrotum with normal scrotal rugae, bilateral cryptorchidism, and normal stretched penile length of approximately 2.8 cm.

## Diagnostic Assessment

### Case 1

Prenatal cell-free DNA screening was consistent with XY and low risk for aneuploidies. Rapid fluorescence in situ hybridization testing confirmed a normal male chromosomal complement with no aneuploidy involving chromosomes 13, 18, 21, X, or Y. Prenatal GS was performed due to the presence of complex cardiac abnormalities. This testing identified a de novo heterozygous likely pathogenic variant located within an intron of *MYRF* (NM_001127392.3): c.46 + 5G > A (Table 1).

**Table 1.** Genetic test results for autosomal dominant variants in the *MYRF* gene

	Variant	Type of variant	Inherited form	Variant classification	Genetic test
**Case 1**	NM_001127392.3: c.46 + 5G > A, p.?	Intronic	De novo	Likely pathogenic	Prenatal genome sequencing
**Case 2**	NM_001127392.2: c.2246A > G, p.(K749R)	Missense	De novo	Likely pathogenic	Prenatal exome sequencing

Pelvic ultrasound obtained on day of life 9 demonstrated a low-lying uterus without visualized gonads. Postnatal biochemical testing during the first week of life was notable for an undetectable serum testosterone level, and low levels of inhibin B and antimüllerian hormone (AMH), suggesting a combined Leydig and Sertoli cell defect consistent with gonadal dysgenesis (Table 2). Gonadotropin levels obtained during mini-puberty at age 6 weeks confirmed very low testosterone concentration (ie, 4.2 ng/dL or 0.14 nmol/L; reference range, 60-400 ng/dL or 2-13.8 nmol/L) along with elevated follicle-stimulating hormone (FSH), consistent with gonadal failure (see Table 2).

**Table 2.** Biochemical evaluation during the newborn period

	Case 1		Case 2	Reference range
	First week of life	Mini-puberty	Mini-puberty	
Testosterone	<2.5 ng/dL (<0.086 nmol/L)	4.2 ng/dL (0.14 nmol/L)	438 ng/dL (15.2 nmol/L)	Mini-puberty range: 60-400 ng/dL (2-13.8 nmol/L)
LH	ND	0.37 mIU/mL	13 mIU/mL	Ultrasensitive assay prepubertal ranges: 0.02-7 mIU/mL
FSH	ND	17 mIU/mL	11 mIU/mL	Ultrasensitive assay prepubertal ranges: 0.16-4.1 mIU/mL
AMH	1.43 ng/mL (10.2 pmol/L)	NA	15.03 ng/mL (107.4 pmol/L)	Male 0-13 d: 15.5-48.7 ng/mL (110-347 pmol/L)
Inhibin B	5 pg/mL	NA	ND	Male <15 d: 68-373 pg/mL (1 pg/mL = 1 ng/L)

Abbreviations: AMH, antimüllerian hormone; FSH, follicle-stimulating hormone; LH, luteinizing hormone; NA, not applicable; ND, no data.

### Case 2

Prenatal cell-free DNA screening was consistent with male fetal sex. Chromosomal microarray from amniocentesis showed a normal male profile (46, XY). ES identified a de novo heterozygous likely pathogenic variant in *MYRF (*NM_001127392.2): c.2246A > G, p.(K749R) (see Table 1). Laboratory evaluation during mini-puberty at age 9 weeks revealed a robust testosterone concentration (438 ng/dL or 15.2 nmol/L; reference range, 60-400 ng/dL or 2-13.8 nmol/L), indicating Leydig cell function. AMH concentration during mini-puberty was on the lower end of normal for a 46, XY individual, indicating possibly some Sertoli cell function (see Table 2). However, both LH and FSH were mildly elevated, suggesting potential underlying gonadal dysfunction (see Table 2).

Scrotal ultrasound on day of life 23 showed ectopic location of the testicles within the lower quadrants of the abdomen. Pelvic ultrasound at age 13 months noted a soft tissue structure posterior to the urinary bladder that was indeterminate (rudimentary uterus, prostate, and/or seminal vesicles).

## Treatment

### Case 1

After extensive review of the clinical and hormonal findings with our DSD team, the family decided on a female sex assignment. The family was also counseled for a potential future risk for gonad malignancy, given intrabdominal dysgenetic testis in an XY individual. The patient underwent a successful repair of the congenital heart defect and was discharged from the hospital at age 2 months.

### Case 2

The patient was assigned male at birth. He underwent surgical repair of the right CDH at day of life 10 and was discharged from the hospital at age 9 months with plans for close follow-up to determine timing of hypospadias repair and orchiopexy.

## Outcome and Follow-up

### Case 1

This patient is followed by multiple specialties including endocrinology and urology with plans for laparoscopy to locate the gonads and possible gonadal biopsy/gonadectomy.

### Case 2

This patient is also followed by multiple specialties with plans to undergo diagnostic laparoscopy, staged hypospadias repair, and orchiopexy with urology around age 18 months.

## Discussion

*MYRF* has been identified as an etiology of 46, XY DSD and OCUGS. These rare conditions present with a wide array of phenotypes that do not show clear genotypic correlation. While syndromic *MYRF*-OCUGS is associated with *MYRF* haploinsufficiency and not clearly associated with a particular protein domain, isolated nanophthalmos (structurally normal small eyes) may result from C-terminal variants that escape nonsense-mediated decay in ocular tissue [8, 9]. In a literature review, Zhang et al [6] identified 17 papers with a total of 31 patients with *MYRF*-related DSD, most with a 46, XY karyotype (one 46, XX twin pair with ovarian dysfunction). Of these, 11 had isolated testicular dysgenesis and 20 had severe cardiopulmonary disease [6]. We present 2 patients with *MYRF*-related DSD found to have different and novel variants demonstrating varying clinical phenotypes in the newborn period.

To our knowledge, the *MYRF* intronic variant carried in patient 1 (c.46 + 5G > A) has not been previously reported and is absent from population databases. Parental genetic testing confirmed this variant as being de novo. This G > A substitution lies at the +5 position of the canonical donor splice site, a critical region for splicing machinery recognition and assembly. Multiple in silico tools predict a marked reduction in splicing efficiency, although SpliceAI predicts minimal effect. Strong evolutionary conservation and reduced promoter activity further supports a likely pathogenic role through combined disruption of splice-site recognition and transcriptional regulation. Functionally, the variant may alter transcription start site usage or cause intron retention, leading to 5′ untranslated region and N-terminal changes that could affect messenger RNA stability and translation, although the precise effect awaits experimental confirmation. Our patient's clinical phenotype—including the presence of müllerian structures and female-appearing external genitalia—overlaps with previously reported cases of *MYRF*-related DSD.

Our second patient carries a de novo likely pathogenic variant in the protein-coding region of *MYRF* (c.2246A > G, p.K749R). This variant is also absent from large population databases and has not been previously reported in the literature. In silico predictions were inconclusive regarding potential splicing effects and suggest no substantial effect on protein structure or function. However, in the absence of RNA or other functional studies, its true biological consequence remains uncertain. A partial loss of MYRF function may underlie the milder GU phenotype in this patient, when compared to case 1.

Pathogenic variants in the *MYRF* gene are thought to impair testis differentiation, leading to gonadal dysgenesis, undervirilization, persistent müllerian structures, and GU anomalies [6]. While we are presenting 2 novel variants and their presentations, it is important to note the variable expressivity known in other *MYRF* variants, including the most reported *MYRF* variant (c.789dup; p.Ser264fs) [6]. This N-terminal frameshift variant is associated with a wide phenotypic spectrum, including CDH, hypoplastic lungs, congenital cardiac disease, ambiguous genitalia, nanophthalmos, and unilateral cryptorchidism [6]. Thus, future patients with these novel variants may also have different presentations. Despite normal testosterone concentrations in infancy (such as patient 2), longitudinal monitoring of gonadal function is important to ensure normal pubertal development and risk of infertility in the future. Even though patient 2 had reassuring testosterone levels during mini-puberty, serum gonadotropin concentrations were mildly elevated, suggesting the need to monitor gonadal function over time. Furthermore, the AMH level concentration was low/normal, which indicates the possibility of persistence of müllerian structures.

These cases illustrate the GU phenotypic variability in *MYRF* variants and underscore the importance of genetic testing for diagnosis. Patient 1 presented with complete sex reversal and was found to have a novel intronic variant, while patient 2 harbored a novel coding variant and presented with GU abnormalities but normal gonadal function during mini-puberty. While there are no defined clinical practice guidelines for this condition, genetic testing enabled clinicians to create a diagnostic approach for patients with identified *MYRF* variants [7]. More information is needed to provide appropriate guidance to families, particularly surrounding the risk of gonadal malignancy, optimal timing for gonadectomy in cases involving sex reversal or potential malignancy, and the long-term outcomes related to gonadal function and fertility. The striking phenotypic differences between these cases underscores the need for continued investigation into the role of *MYRF* in sexual development and its broader implication in human disease.

## Learning Points

- Variants in *MYRF* are a rare etiology of XY differences of sex development in addition to ocular, pulmonary, and cardiac malformations.
- GS enables the identification of novel variants that may have functional and phenotypic significance, including intronic variants that might otherwise go undetected.
- There is a wide phenotypic spectrum of *MYRF-*related disorders and there does not appear to be a clear genotype-phenotype association for *MYRF*-OCUGS, though variants affecting the C-terminus of the protein appear to be associated with isolated nanophthalmos.

## Contributors

All authors made individual contributions to authorship. A.D., P.C., M.K., M.V., and S.M. were involved in the diagnosis and management of one or both cases. C.M. was involved in the discussion section and interpretation of genetic reports. All authors reviewed and approved the final draft.

## Data Availability

Data sharing is not applicable to this article as no datasets were generated or analyzed during the current study.

## Abbreviations

AMH: antimüllerian hormone
CDH: congenital diaphragmatic hernia
DSD: differences of sex development
ES: exome sequencing
FSH: follicle-stimulating hormone
GS: genome sequencing
GU: genitourinary
LH: luteinizing hormone
MYRF: myelin regulatory factor
NGS: next-generation sequencing
OCUGS: ophthalmic cardiac urogenital syndrome
